# Aerobic Exercise Promotes Hippocampal Neurogenesis and Ameliorates Cognitive Dysfunction Induced by Unilateral Labyrinthectomy

**DOI:** 10.1002/cns.70773

**Published:** 2026-02-03

**Authors:** Zhanghong Zhou, Xixi Yu, E. Tian, Zhaoqi Guo, Jingyu Chen, Jiaqi Guo, Shiyu Shi, Wandi Xu, Ni Zhai, Caijuan Qiao, Yuejin Zhang, Jun Wang, Yisheng Lu, Sulin Zhang

**Affiliations:** ^1^ Department of Otorhinolaryngology, Union Hospital, Tongji Medical College Huazhong University of Science and Technology Wuhan China; ^2^ Department of Rehabilitation, Liyuan Hospital of Tongji Medical College Huazhong University of Science and Technology Wuhan China; ^3^ Department of Physiology, School of Basic Medicine Huazhong University of Science and Technology Wuhan China; ^4^ School of Life Sciences and Technology Tongji University Shanghai China; ^5^ Department of Otorhinolaryngology, Head and Neck Surgery, the First Affiliated Hospital, Jiangxi Medical College Nanchang University Nanchang China

**Keywords:** aerobic exercise, cognitive impairment, hippocampal neurogenesis, neuroinflammation, vestibular dysfunction

## Abstract

**Aims:**

Vestibular dysfunction is strongly linked to cognitive impairment and dementia risk, yet its underlying mechanisms remain unclear, hindering treatment development. As a primary physical therapy intervention, aerobic exercise improves cognitive dysfunction. Thus, we investigated its potential to reverse cognitive deficits after vestibular dysfunction and explored the neurobiological mechanisms involved.

**Methods:**

Behavioral experiments were conducted to assess the cognitive impairment induced by unilateral labyrinthectomy (UL). Immunofluorescence staining was performed to assess neural stem cell proliferation and newborn neuron maturation in the hippocampal dentate gyrus. Aerobic exercise was applied as an intervention to examine its effects on cognitive and neurogenic recovery. RNA sequencing followed by qRT‐PCR was used to characterize transcriptional changes. Pharmacological manipulation is applied to demonstrate the necessity and sufficiency of key candidate genes in mediating the exercise‐induced effects.

**Results:**

UL induced significant cognitive dysfunction and hippocampal neurogenesis inhibition, whereas aerobic exercise intervention reversed these impairments. Transcriptomic profiling showed that aerobic exercise normalized UL‐disrupted gene expression, with enrichment observed in MHC class I/II–related (H2‐K1, H2‐Eb1, CD74) and neurodevelopmental (Cntn5, Mid1) pathways. Pharmacological experiments established causality: inhibition of neuroinflammatory signaling with AZD1480 enhanced neurogenesis and improved cognition after UL, whereas LPS‐mediated activation of inflammation attenuated the beneficial effects of aerobic exercise.

**Conclusion:**

These findings provide a novel non‐pharmacological strategy for cognitive rehabilitation, as well as early cognitive screening and exercise rehabilitation intervention for patients with vestibular disorders.

## Graphical abstract

UL induced significant spatial and non‐spatial cognitive dysfunction and hippocampal neurogenesis inhibition, whereas aerobic exercise intervention effectively reversed these impairments. Transcriptomic profiling and pharmacological evidence identified MHC class I/II–related pathways (H2‐K1, H2‐Eb1, CD74) as key mediators of the restorative effects of aerobic exercise.

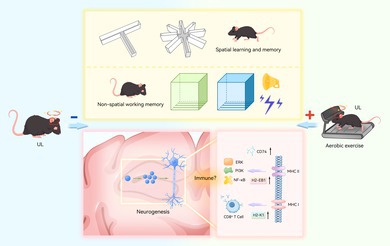

**Abbreviations**
AHN: adult hippocampal neurogenesis
BVL: bilateral vestibular loss
CD74: CD74 molecule
Cntn5: contactin 5
DEGs: differentially expressed genes
DG: dentate gyrus
GO: gene ontology
H2‐Eb1: histocompatibility 2‐class II antigen E beta
H2‐K1: histocompatibility 2‐K1
KEGG: Kyoto Encyclopedia of Genes and Genomes
LPS: lipopolysaccharide
LTP: long‐term potentiation
Mid1: Midline 1
NSC: neural stem cells
OFT: open‐field test
qRT‐PCR: quantitative real‐time PCR
RAM: radial 8‐arm maze
UL: unilateral labyrinthectomy
UVL: unilateral vestibular loss
VH: vehicle

## 1 Introduction

The vestibular system plays a pivotal role in numerous functions, ranging from gaze stabilization and postural control to high‐level cortical processes. Approximately 35.4% of adults over the age of 40 suffer from vestibular dysfunction, with its prevalence increasing significantly with advancing age [1]. In addition to balance disorders, individuals with peripheral vestibular dysfunction may also exhibit deficits in various cognitive domains, such as spatial navigation, memory, and attention [2, 3]. Moreover, several studies have reported that vestibular loss is associated with an increased risk of developing dementias such as Alzheimer's disease [4, 5, 6]. While accumulating empirical investigations have demonstrated an association between vestibular pathology and cognitive deficits, critical knowledge gaps persist regarding both the precise pathophysiological mechanisms mediating this connection and the development of evidence‐based therapeutic interventions. Subsequently, targeted rehabilitative strategies specifically addressing vestibular‐induced cognitive impairment remain conspicuously underexplored.

The hippocampus is an important part of the limbic system that has essential roles in memory, navigation, and cognition [7]. Vestibular information can be transmitted to the parietal lobe through the thalamus and further to the entorhinal cortex or hippocampus to complete the recognition of environmental spatial information [8]. Several reports suggested spatial memory deficits and impaired hippocampal plasticity following complete unilateral or bilateral vestibular loss in rats [9, 10, 11]. Our previous research also indicates that unilateral vestibular dysfunction causes persistent cognitive deficits in mice, which are associated with altered neuronal activation and gene expression in the hippocampus [11]. Consistently, patients with chronic bilateral vestibular loss present with a 16.9% decrease in hippocampal volume, which suggests altered neuronal cytoarchitecture or even a decrease in hippocampal neural stem cell and neurogenesis [12, 13]. Thus, cognitive impairment following vestibular dysfunction is strongly associated with pathological changes in the hippocampus.

Aerobic exercise has emerged as a potent non‐pharmacological intervention with multi‐system benefits, demonstrating particular efficacy in neurological rehabilitation and cognitive preservation [14, 15, 16, 17]. Extensive clinical evidence supports its role in mitigating cognitive decline associated with aging and Alzheimer's disease through mechanisms ranging from enhanced angiogenesis and neurovascular remodeling to modulation of inflammatory pathways and neurotrophic factor regulation [18, 19, 20, 21]. The pro‐cognitive effects of exercise are particularly evident in hippocampal plasticity adult hippocampal neurogenesis (AHN), the continuous generation of neurons from neural stem cells (NSC) in the dentate gyrus (DG) [22, 23]. Both acute (7‐day) and chronic (4‐week) exercise regimens in rodents induce NSC proliferation and neuronal survival, with running wheel access enhancing synaptic plasticity markers like long‐term potentiation while improving spatial memory and pattern separation abilities [24, 25, 26]. Notably, these neurogenic changes correlate with functional improvements across multiple cognitive domains, including cognitive flexibility and memory precision, suggesting AHN serves as a critical mediator of exercise‐induced cognitive preservation [27, 28, 29, 30]. The convergence of human neuroimaging data showing exercise‐induced hippocampal volume restoration with preclinical evidence of amyloid pathology reduction establishes aerobic exercise as a multimodal therapeutic strategy targeting both structural and functional aspects of brain health [31].

On these bases, we propose a hypothesis that vestibular lesions inhibit hippocampal neurogenesis and impair cognitive function, while aerobic exercise promotes hippocampal neurogenesis and contributes to cognitive rehabilitation following vestibular dysfunction. Thus, we comprehensively evaluated the cognitive function and hippocampal neurogenesis after UL in mice treated with and without aerobic exercise. We aim to provide translational evidence that aerobic exercise may serve as a promising non‐pharmacological intervention to enhance hippocampal neurogenesis and accelerate cognitive rehabilitation in patients with vestibular disorders, offering new perspectives for developing neurorehabilitation strategies.

## 2 Materials and Methods

### 2.1 Animals

Experimental procedures involving mice were approved by the Animal Welfare Committee of Huazhong University of Science and Technology (No. 3971). All experiments were conducted in strict accordance with the committee guidelines. C57BL/6J mice (8 weeks old) were obtained from the Experimental Animals Center of Tongji Medical College, Huazhong University of Science and Technology. To minimize variability associated with cyclical hormonal fluctuations in females, only male mice were used in this study. Animals were group‐housed (≤ 5 per cage) under controlled environmental conditions (temperature: 22°C–24°C; humidity: 55%–80%) with a 12‐h light/dark cycle and provided ad libitum access to food and water.

### 2.2 Unilateral Labyrinthectomy

Male mice (22–25 g) were randomly allocated to the UL or sham surgery groups. In the UL group, mice were anesthetized with 1% sodium pentobarbital (35 mg/kg, i.p.). A postauricular incision was made to expose the middle ear structures. The tympanic membrane was then punctured using fine forceps, and the malleus, incus, and stapes were carefully removed. The stapedial artery was then cauterized using an electrocautery device, and the vestibular window was widened with a probe to allow deep puncture of the labyrinth, ensuring complete destruction. Following this, a small amount of anhydrous ethanol was injected to further damage the labyrinth, and a gelatin sponge was placed in the vestibular window. The incision was then sutured closed. In the sham surgery group, the tympanic membrane was perforated, and the ossicles were removed, but the vestibular structures and labyrinth remained intact.

### 2.3 Aerobic Exercise

During the first week, mice in the aerobic exercise groups performed adaptive running training in individual treadmill lanes (FT‐200, Taimeng, China) at a speed of 5 m/min for 45 min per session. Over the following 3 weeks, the running protocol was adjusted to 5 m/min for 10 min, 8 m/min for 30 min, and 5 m/min for 10 min per session to minimize stress‐induced inhibition of hippocampal neurogenesis [32]. Mice in the static groups were placed on the stationary treadmill for the same duration.

### 2.4 Behavioral Analysis

Behavioral analysis was conducted by investigators blinded to the group assignments. All tests were conducted during the light phase. Prior to testing, mice were acclimated by handling for a minimum of 5 min, twice daily, for three consecutive days. No significant differences in body weight or whisker number were observed between groups.

### 2.5 Open‐Field Test (OFT)

Mice were placed at the center of a rectangular chamber (45 × 45 × 45 cm), and their movement was monitored for 5 min using an automated video tracking system (Supermaze 2.0, Softmaze, China). To mitigate the impact of olfactory cues, the apparatus was cleaned with 75% alcohol following each trial. The total distance traveled during each session was documented.

### 2.6 T‐Maze

The apparatus consisted of an opaque acrylic T‐maze (50 cm long, 10 cm wide, and 25 cm high), with a camera mounted above and connected to a video tracking system (Supermaze 2.0, Softmaze, China). To minimize stress and promote acclimatization, mice were placed in the testing room 24 h before the experiment. The experimental procedure was divided into two distinct phases: a learning phase and a testing phase. During the learning phase, only one arm of the T‐maze (either the left or the right) was open, allowing the mouse to freely explore the maze for 5 min before returning to its home cage. Following a 30‐min interval, during the testing phase, both arms of the T‐maze were open, and the mouse was permitted to explore the maze freely for another 5 min. The percentage of entries into and the total exploration time in the closed arms during the acquisition phase were analyzed.

### 2.7 Radial 8‐Arm Maze (RAM)

The RAM was composed of eight transparent organic plastic arms equally spaced around an octagonal central platform. Before the training sessions, the animals' body weight was maintained at 85% of their free‐feeding weight, with water available ad libitum. To facilitate acclimatization to the RAM, all arms were baited with one food pellet placed at the distal terminal, allowing mice to explore the maze for 10 min. This process was conducted twice daily over three consecutive days. Subsequently, the mice underwent 20 testing sessions during 10 consecutive days, with two sessions per day spaced by a minimum of 2 h. During each testing session, the animals were allowed to visit all 8 baited arms within 5 min. A repetition error was recorded when a mouse re‐entered a previously visited arm, while an omission error was noted if a mouse failed to visit an arm.

### 2.8 Contextual and Tone‐Cued Fear Conditioning

The shock‐associated training context (Context A) and the analogous non‐shock context (Context B) had the same exposed stainless‐steel grid floor and ceiling. Context B differed from Context A only in that it included four plastic wall inserts. To maintain cleanliness between trials, a non‐alcoholic antiseptic solution was utilized to disinfect the grids. On the first day, the mice were permitted to explore the conditioning chamber (Context A; 29 × 29 × 24 cm; H10‐11M‐TC, Coulbourn Instruments) for 180 s before a tone presentation. The tone lasted 30 s at 80 dB and 5 kHz. During the final second of the tone, a 0.75 mA foot shock was administered, co‐terminating with the tone. The tone‐shock pairing was repeated three times, with an intertrial interval of 30 s. Following the conditioning session, the mice were returned to their home cages. The freezing response during the final tone‐shock pairing was quantified to assess the extent of learning. The following day, the mice were placed in context A for 5 min without administering shock or tone to evaluate the contextual fear memory by quantifying the freezing response. On the third day, the tone‐cued fear memory was assessed by exposing the mice to Context B while presenting a tone lasting 30 s at 80 dB and 5 kHz without shock. The freezing response was analyzed during this time. If no mouse movement was detected for more than 2 s, its behavior was counted as “freezing”, and freezing levels were recorded by a video camera (Freezeframe, Coulbourn Instruments) [33].

### 2.9 Stereotaxic Viral Injection

Adult mice were anesthetized with 1% pentobarbital sodium (35 mg/kg, i.p.) and head‐fixed in a stereotaxic device (68,025, RWD Life Science, China). After skin disinfection, the skin on the mice's heads was cut with tissue scissors to expose the cranial bones. A skull drill was used to make a small hole in the skull bone and the viruses were injected (300 nL per side, 40 nL/min) into the ipsilateral hippocampus with a glass pipette (tip size, ~20 μm) at the following coordinates relative to bregma: anteroposterior, −2.06 mm; dorsoventral, −2.0 mm; and mediolateral, ±1.5 mm. The glass pipette was left in place for 10 min before being gradually withdrawn. The skin was sutured and sterilized with iodophors. The animals were then subjected to behavioral tests 3 weeks after virus injection, and the brains were sectioned afterward to verify the infusion sites. Data were excluded if the GFP expression was misdirected.

### 2.10 BrdU Injection

Mice were administered 5′‐Bromo‐2′‐Deoxyuridine (BrdU; B5002, Sigma) in saline via intraperitoneal injection at a dose of 100 mg/kg, once daily for five consecutive days. To avoid labeling the acute burst of progenitor proliferation induced by surgery or stimulation, we administered BrdU during the late phase of the intervention to more accurately capture the cell populations most relevant to exercise‐responsive neurogenesis. Animals were sacrificed 24 h after the final BrdU injection.

### 2.11 Immunofluorescence Staining

Mice were anesthetized using 1% pentobarbital sodium (35 mg/kg, i.p.) and perfused transcardially with 0.1 M PBS, followed by 4% paraformaldehyde in 0.1 M PBS. The brains were then removed and fixed in 4% paraformaldehyde at 4°C overnight. After dehydration in 30% sucrose, brains were frozen in OCT medium (TissueTek, Sakura, Japan) and sliced into 30 μm coronal sections using a cryostat (HM550, Thermo Scientific). After three rinses with PBS, brain sections were rinsed in blocking buffer (PBS with 0.3% Triton X‐100 containing 10% goat serum and 3% BSA) for 60 min at room temperature. Sections were incubated overnight at 4°C in blocking buffer containing rat anti‐BrdU antibody (ab6326, Abcam; 1:300 dilution), rabbit anti‐Ki67 antibody (ab15580, Abcam; 1:500 dilution), and rabbit anti‐DCX antibody (13925‐1‐AP, Proteintech; 1:300 dilution). After three washes with PBST (PBS containing 0.05% Tween‐20), sections were incubated with the appropriate fluorophore‐conjugated secondary antibody (goat anti‐rabbit IgG conjugated to Alexa Fluor 488, AS039, ABclonal; 1:300 dilution) in blocking buffer for 1 h at room temperature. After a further three washes with PBST, the sections were mounted on glass slides using a mounting medium containing DAPI (BL739A, Biosharp, China). Confocal microscopy images were captured using a Zeiss LSM 780 microscope (Germany). For cell counting, every sixth section of consecutive sections was selected for staining, resulting in five sections per mouse being analyzed. Each experimental group consisted of five mice. This approach was adopted to minimize bias that could arise from the selection of sections biased towards the anterior or posterior axis. Positive cell counts were performed in the granule cell layers and hilus (polymorphic layer) of the DG at consistent anatomical locations in each brain section. The individuals performing the cell counts were blinded to the group assignments during the analysis.

### 2.12 Sholl Analysis

Sholl analysis was performed as previously described [32]. The total dendritic branches in one GFP+ neuron in the DG were scanned by means of an Olympus Fluoview FV1000 (Olympus, Japan). The dendritic branches were analyzed using the ImageJ Sholl Analysis Plugin, with the center of each concentric circle defined as the center of the cell soma. Five mice were selected for each experimental group and five brain sections were analyzed per mouse. Within each section, 8 to 10 cells in the DG were analyzed and counted.

### 2.13 Dissection of Hippocampal Dentate Gyrus

The DG was isolated as described previously [34]. Briefly, mice were anesthetized with 1% sodium pentobarbital (35 mg/kg, i.p.), and the brain was removed and immediately placed in ice‐cold PBS. The brain was sectioned along the longitudinal fissure in a petri dish containing ice‐cold PBS, and the regions posterior to the lambda (midbrain, hindbrain, and cerebellum) were carefully excised. Subsequently, the medial side of the cerebral hemisphere was oriented upward, and the diencephalon was meticulously removed under the guidance of a dissection microscope, exposing the medial side of the hippocampus. A sharp needle tip was inserted into each side of the DG and advanced along the septo‐temporal axis of the hippocampus to facilitate the isolation of the DG.

### 2.14 RNA Extraction and Sequencing

Total RNA was extracted from the DG using the TRIzol reagent protocol (Life Technologies, California, USA). RNA concentration and purity were assessed using a NanoDrop 2000 spectrophotometer (Thermo Fisher Scientific, Wilmington, DE), and RNA integrity was assessed using the RNA Nano 6000 Assay Kit on the Agilent Bioanalyzer 2100 system (Agilent Technologies, CA, USA). For each sample, 1 μg of RNA was used for library preparation. RNA sequencing (RNA‐seq) libraries were constructed using the Hieff NGS Ultima Dual‐mode mRNA Library Prep Kit for Illumina (Yeasen Biotechnology Co. Ltd.), according to the manufacturer's instructions. Unique index codes were added to each sample to facilitate the assignment of sequence data. Specifically, mRNA was isolated from total RNA using poly‐T oligo‐attached magnetic beads. First‐strand cDNA was then synthesized, followed by second‐strand cDNA synthesis. The resulting overhangs were converted to blunt ends by exonuclease and polymerase activities. After adenylating the 3′ ends of the DNA fragments, a NEBNext adaptor with a hairpin loop structure was ligated to the fragments in preparation for hybridization. The library fragments were purified using the AMPure XP system (Beckman Coulter, Beverly, USA). Subsequently, 3 μL of USER enzyme (NEB, USA) was used with size‐selected, adaptor‐ligated cDNA at 37°C for 15 min, followed by incubation at 95°C for 5 min before qRT‐PCR. Polymerase chain reaction (qRT‐PCR) was performed using Phusion High‐Fidelity DNA Polymerase, universal qRT‐PCR primers, and Index (X) primers. qRT‐PCR products were re‐purified using the AMPure XP system, and library quality was assessed using the Agilent Bioanalyzer 2100 system. Libraries were sequenced on the Illumina NovaSeq platform according to the manufacturer's protocol, generating 150 bp paired‐end reads. Raw data analysis was performed using BMKCloud (www.biocloud.net). DEGs between comparison groups were identified using standardized data processing and screening. Each group consisted of four mice, with default parameters set to an FDR of 0.01 and a fold change (FC) of 2. Functional annotation, classification, and pathway analysis of DEGs were performed using the KEGG database.

### 2.15 Quantitative Real‐Time PCR (qRT‐PCR)

DG tissue samples were isolated on ice and immediately stored at −80°C. Total RNA was extracted using RNAiso Plus (code 9108, TaKaRa). RNA concentration and purity were assessed using NanoDrop2000C spectrophotometry (Thermo Scientific). One microgram of RNA was reverse transcribed to complementary DNA (cDNA) using HiScript II Q RT SuperMix for quantitative reverse transcription PCR (qRT‐PCR) with a genomic DNA (gDNA) wiper (R22301, Vazyme, Nanjing, China). qRT‐PCR was performed on a BioRad CFX384 Real‐Time System using ChamQ SYBR qRT‐PCR Master Mix (Q311‐02, Vazyme, Nanjing, China) according to the manufacturer's protocol. The abundance of different transcripts was assessed in triplicate. The primer sequences used for amplification are listed in Table 1.

**TABLE 1:** Primer sequences of genes.

Gene name	Gene ID	Primer sequences
Lyz2	ENSMUSG00000069516	F5′‐ATGGAATGGCTGGCTACTATGG‐3
		R5′‐ACCAGTATCGGCTATTGATCTGA‐3
5CD74	ENSMUSG00000024610	F5′‐AGTGCGACGAGAACGGTAAC‐3
		R5′‐CGTTGGGGAACACACACCA‐3
Cntn5	ENSMUSG00000039488	F5′‐GGCGGTTCATATCCCAAGAGA‐3
		R5′‐ACTGAGCACGCGAGCATTT‐3
Glp2r	ENSMUSG00000049928	F5′‐AGGAGACAGTTCAGAAGTGGG‐3
		R5′‐GCCAGCACACGTACTTATCAA‐3
Slc11a1	ENSMUSG00000026177	F5′‐GCAGGCCCAGTTATGGCTC‐3
		R5′‐CAGGCTGAATGTACCCTGGTC‐3
Des	ENSMUSG00000026208	F5′‐GTGGATGCAGCCACTCTAGC‐3
		R5′‐TTAGCCGCGATGGTCTCATAC‐3
Gins2	ENSMUSG00000031821	F5′‐GAGGTGGAGTTTTTGGCCGAA‐3
		R5′‐GGTAAGCCGGGGTTGAAGG‐3
Clec1a	ENSMUSG00000033082	F5′‐ATGCAGGCCAAATACAGCAG‐3
		R5′‐CCAGAATACAGGCTTATGGTGGT‐3
Mid1	ENSMUSG00000035299	F5′‐CTGTGACGGCACCTGTCTC‐3
		R5′‐AAACGGCTGACTGTTGGTCTT‐3
H2‐K1	ENSMUSG00000061232	F5′‐CAGGTGGAGCCCGAGTATTG‐3
		R5′‐CGTACATCCGTTGGAACGTG‐3
H2‐Eb1	ENSMUSG00000060586	F5′‐GCGGAGAGTTGAGCCTACG‐3
		R5′‐CCAGGAGGTTGTGGTGTTCC‐3
Hba‐a1	ENSMUSG00000069919	F5′‐CACCACCAAGACCTACTTTCC‐3
		R5′‐CAGTGGCTCAGGAGCTTGA‐3

### 2.16 The JAK1/JAK2 Inhibitor AZD1480 Attenuates Neuroinflammation

At 2 weeks after UL induction, mice received daily oral gavage of either AZD1480 (HY‐10193, MCE) at 25 mg/kg or a vehicle (VH) control consisting of 0.1% dimethyl sulfoxide (DMSO; HY‐Y0320, MCE) [35]. Depending on the experimental endpoint, mice were either used for behavioral assays or sacrificed 2 weeks post‐dosing. Upon sacrifice, animals underwent transcardial perfusion with ice‐cold phosphate‐buffered saline (PBS, pH 7.4), which was followed by fixation using a 4% paraformaldehyde solution in preparation for immunofluorescence staining.

### 2.17 LPS Induces Neuroinflammatory Responses

Two weeks after UL and running, mice were intraperitoneally injected daily with 0.25 mg/kg lipopolysaccharide (LPS) (L2880, Sigma) or saline as a vehicle control [36]. Based on analytical requirements, mice were either subjected to behavioral tests or euthanized 2 weeks after administration. Upon sacrifice, transcardial perfusion was performed using ice‐cold phosphate‐buffered saline (PBS, pH 7.4), followed by fixation with a 4% paraformaldehyde solution for subsequent immunofluorescence staining.

### 2.18 Statistical Analysis

All statistical analyses were performed using GraphPad Prism 8.0 (GraphPad Software, USA). The normality of data distribution was assessed using the Shapiro–Wilk test. Data conforming to a normal distribution were analyzed using the two‐tailed Student's *T*‐test, one‐way ANOVA followed by Tukey's post hoc test, or two‐way ANOVA with Bonferroni post hoc test. All data are presented as the mean ± standard error of the mean (SEM). Non‐normally distributed data were analyzed using the Mann–Whitney *U* test. Statistical differences were considered significant if *p* < 0.05.

## 3 Results

### 3.1 Cognitive Impairment 28 Days After UL

**FIGURE 1:**
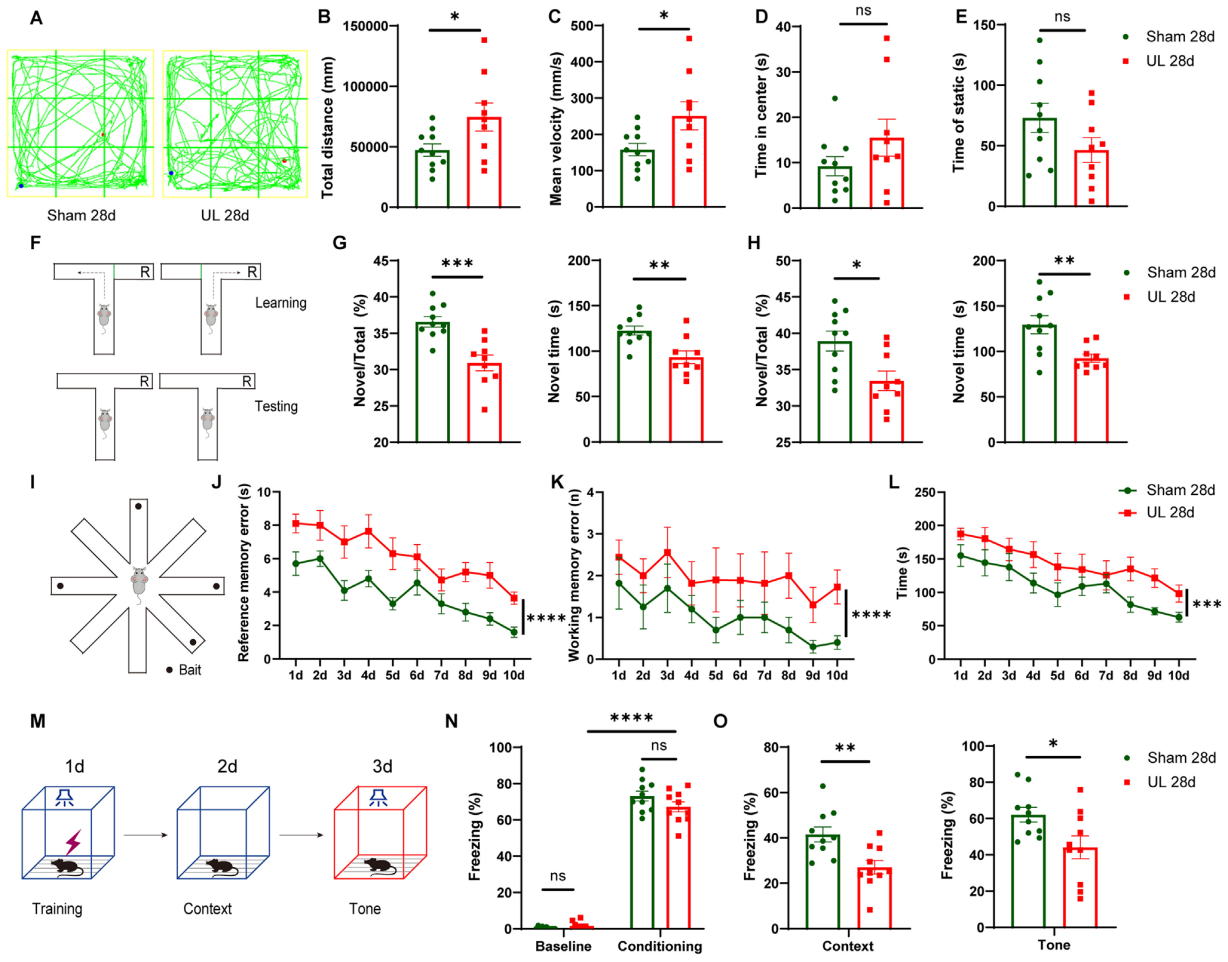
Cognitive impairment in mice at 28 days after UL. (A) Representative movement trajectories were recorded in an open field, wherein each mouse was permitted to explore freely for a duration of 5 min. (B) Total locomotion distance increased in UL group (two‐tailed Student's *t*‐test, *t* = 2.237, *p* = 0.0390). (C) Average speed increased in UL group (two‐tailed Student's *t*‐test, *t* = 2.275, *p* = 0.0361). (D) No significant difference in time spent in the center of the open field between the two groups (two‐tailed Student's *t*‐test, *t* = 1.668, *p* = 0.1135) (E) A comparison of the total time spent stationary in the open field between the sham and UL groups revealed no significant difference (two‐tailed Student's *t*‐test, *t* = 1.421, *p* = 0.1733) (*n* = 10 and 9 for Sham and UL mice in OFT). (F) The following schematic illustrates the experimental design of the T‐maze, delineating the phases of learning and testing. During the learning phase, only one arm (left or right) was exposed whereas in the testing phase, both arms were made accessible. (G, H) The working memory impairment in the UL group was evident through the total number of entries into and the time spent in the novel arm. Right arm (G) was set as the novel arm (two‐tailed Student's *t*‐test, *t* = 4.441, *p* = 0.0004 for the percentage of novel arms; two‐tailed Student's *t*‐test, *t* = 3.447, *p* = 0.0031 for the total time of the novel arms). Left arm (H) was set as the novel arm (two‐tailed Student's *t*‐test, *t* = 2.501, *p* = 0.0236 for the percentage of novel arms; two‐tailed Student's *t*‐test, *t* = 2.952, *p* = 0.0094 for the total time of the novel arms, *n* = 10 and 9 for Sham and UL mice in T maze). (I) Schematic diagram of the 8‐arm maze experiment design. During the 10‐day training period, all eight arms were accessible. Food pellets were placed at the ends of four randomly selected arms, with two trials per day. (J) The number of errors in the reference memory markedly increased in the UL group (two‐way ANOVA, *F*_(1,179)_ = 59.23, *p* < 0.0001). (K) The number of errors in the working memory increased in the UL group (two‐way ANOVA, *F*_(1,179)_ = 18.06, *p* < 0.0001). (L) Total time to complete tasks significantly increased in UL group (two‐way ANOVA, *F*_(1,20)_ = 20.69, *p* = 0.0002, *n* = 10/group). (M) Schematic diagram of the fear conditioning experimental design. On Day 1, mice received foot shocks paired with a tone in Context A. On Day 2, mice were exposed to Context A without shocks or tones to test contextual fear conditioning. On Day 3, mice were placed in a similar Context B with the tone but no shocks to test tone‐cued fear conditioning. (N) Throughout the training, Sham and UL mice froze at a comparable level during the trace interval (*n* = 10/group, two‐way ANOVA, *F*_(1,36)_ = 1217, *p* < 0.0001). (O) Contextual and tone‐cued fear conditioning were impaired in UL mice (*n* = 10/group, unpaired *T*‐test, *p* = 0.0044 for contextual fear conditioning and *p* = 0.0275 for tone‐cued fear conditioning). *p < 0.05, **p < 0.01, ***p < 0.001, ****p < 0.0001.

To minimize the risk of false positives in subsequent cognitive behavioral assessments due to UL‐induced acute locomotor impairments, we evaluated hyperactivity and anxiety‐like behaviors using the OFT 28 days after UL (Figure 1A). Compared with sham‐operated mice, UL mice exhibited a significant increase in total movement distance (Figure 1B) and higher average movement speed (Figure 1C). No significant differences were observed between these two groups in terms of total static time and the duration spent in the center of the open field (Figure 1D,E). These findings suggest that locomotor ability was not compromised and that there was no evidence of anxiety‐like behavior [12, 37].

Our subsequent study focused on the assessment of cognitive behaviors. The T‐maze task was employed to assess short‐term (30 min) spatial memory in UL mice (Figure 1F). To circumvent the occurrence of false positives because of directional bias, training sessions were conducted with either the left or right arm of the maze being closed alternately. UL mice exhibited significantly fewer entries and spent less time in the novel arm compared with sham controls (Figure 1G,H). For spatial working memory, as evaluated by the RAM test (Figure 1I), both groups demonstrated a decrease in error entries and total task completion time across days, indicating that all mice were able to learn the task. However, UL mice made a significantly higher number of wrong arm entries (Figure 1J,K), which likely contributed to the increased total task completion time (Figure 1L), suggesting the spatial working memory deficit in UL mice. For fear conditioning test, the freezing levels during the third tone‐shock pairing conditioning phase were comparable between the sham and UL groups (Figure 1M,N), suggesting that the learning ability was normal in UL group. However, spatial and tone‐cued long‐term memories were impaired in UL group, manifested by contextual and tone‐cued fear conditioning tests (Figure 1O).

### 3.2 Inhibition of DG Neurogenesis in Mice at 28 Days After UL

**FIGURE 2:**
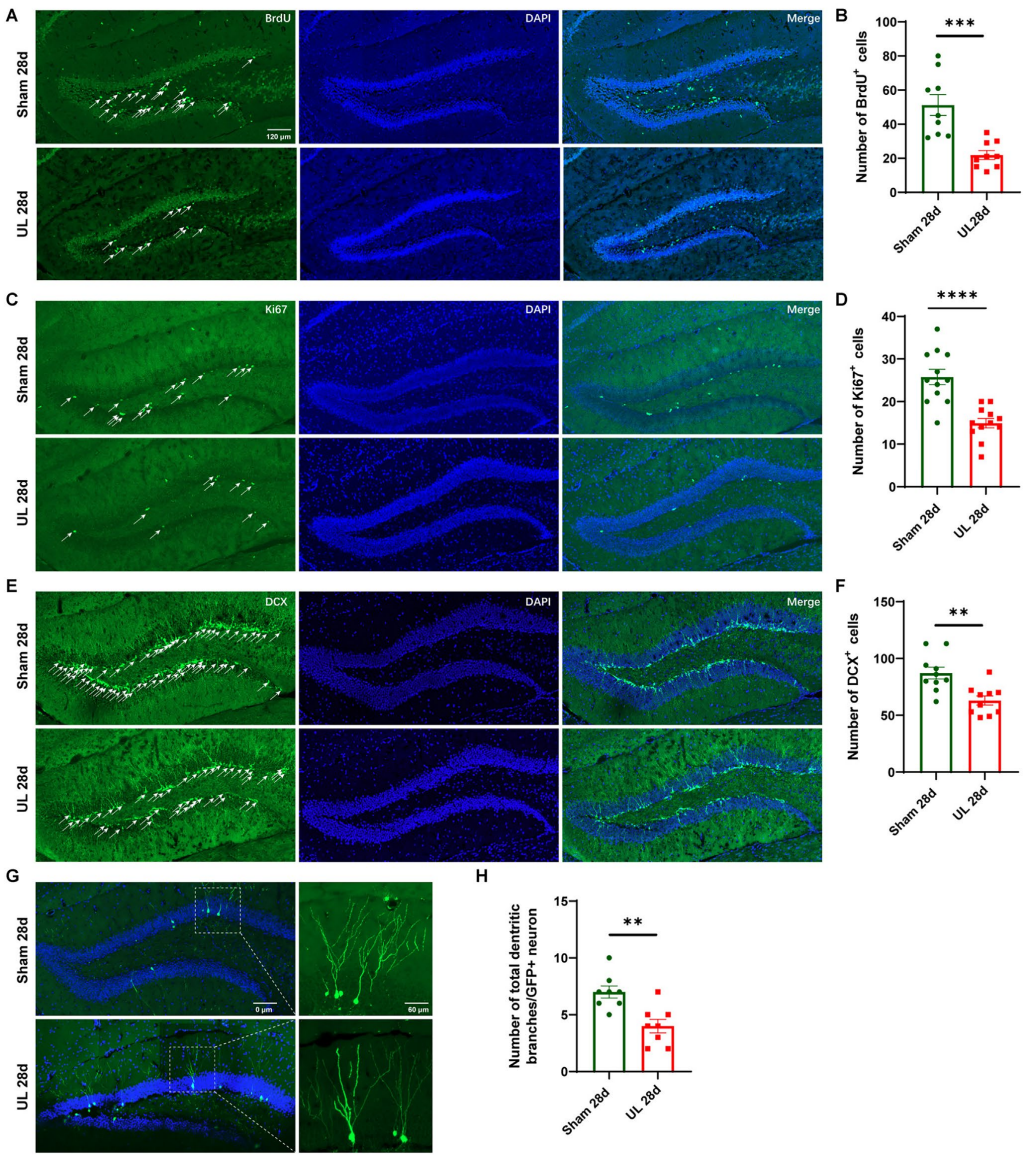
Decreased adult neurogenesis in the DG of UL mice. (A) Representative photomicrographs showing BrdU and DAPI+ cells in the DG. (B) The number of BrdU+ cells in the DG decreased in UL mice (ten mice per group and five sections per mouse, unpaired *T*‐test, *t* = 4.401, *p* = 0.0004). (C) Representative photomicrographs showing Ki67+ and DAPI+ cells in the DG. (D) The number of Ki67+ cells in the DG decreased in UL mice (ten mice per group and five sections per mouse, unpaired *T*‐test, *t* = 5.163, *p* < 0.0001). (E) Representative photomicrographs showing DCX and DAPI+ cells in the DG. (F) The number of DCX+ cells in the DG decreased in UL mice (ten mice per group and five sections per mouse, unpaired *T*‐test, *t* = 3.691, *p* = 0.0017). (G) Representative photomicrographs of the dendritic branches of GFP+ cells in the DG. GFP+ cells were labeled with retrovirus pROVEF1a‐EGFP, which was injected in the DG. (H) The total number of dendritic branches of one GFP+ neuron decreased in UL mice (eight mice per group, five sections per mouse, and 8–10 cells per section, unpaired *T*‐test, *t* = 3.742, *p* = 0.0022).

In alignment with the observed cognitive deficits, UL mice exhibited a significant reduction in neural stem cell proliferation within the DG, as demonstrated by quantitative analysis of BrdU incorporation (Figure 2A,B), as well as Ki67‐ and DCX‐positive cells (Figure 2C–F). To fully characterize the maturation process of neural stem cells in the DG, we used a retroviral labelling assay in which newborn neurons were specifically labeled with pROV‐EF1a‐EGFP by microinjection into the DG immediately after UL surgery in mice, and the maturation of neural stem cells was assessed 28 days later. Quantitative morphological analysis using the Sholl method revealed a significant reduction in total dendritic branching complexity compared to controls (Figure 2G,H). These findings indicate that UL leads to reduced proliferation and maturation of NSCs in the DG, suggesting a potential causal relationship between impaired neurogenic processes and cognitive dysfunction.

### 3.3 Aerobic Exercise Improves Cognitive Function in Mice After UL

**FIGURE 3:**
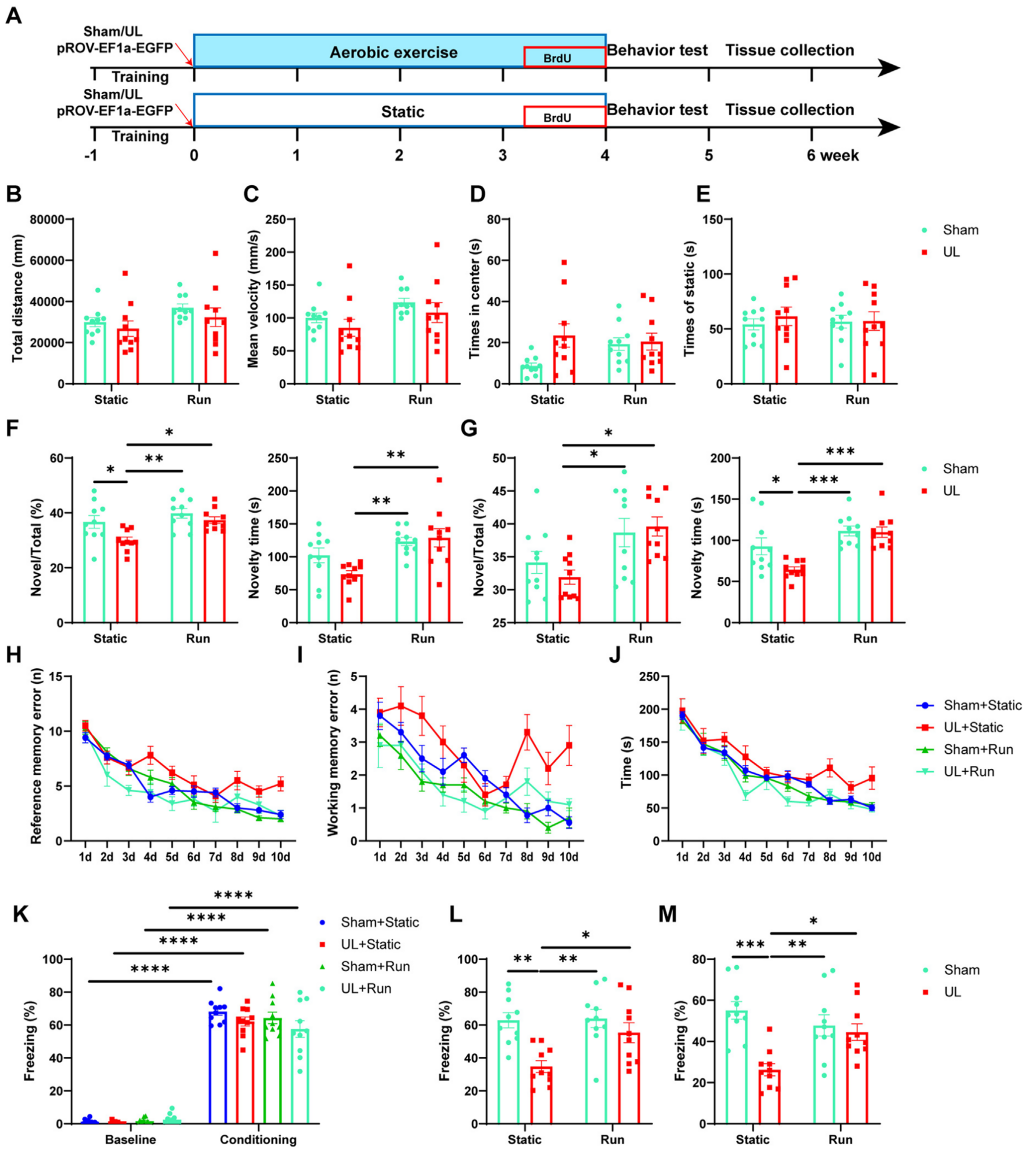
Cognitive dysfunction in mice after UL was improved by aerobic exercise (A) Schematic diagram of the experimental design of an exercise intervention in UL mice. (B, C) In the open field test, there were no significant differences in total movement distance and average speed among the four groups of mice: Sham + Static, Sham + UL, Run + Static, and Run + UL (two‐way ANOVA: *F*_(1,36)_ = 0.04837, *p* = 0.8272 and *F*_(1,36)_ = 0.0009785, *p* = 0.9752). (D, E) There were no significant intergroup differences in terms of total immobility time and the duration spent in the central zone of the open field for the Sham + Static, Sham + UL, Run + Static, and Run + UL groups (two‐way ANOVA: *F*_(1,36)_ = 3.000, *p* = 0.0918 and *F*_(1,36)_ = 0.2262, *p* = 0.6372, *n* = 10/group in OFT). (F, G) Aerobic exercise ameliorated the UL‐induced working memory impairment, manifested by the total entry to and time spent in the novel arm. Right arm (F) was set as the novel arm (two‐way ANOVA: *F*_(1,36)_ = 1.616, *p* = 0.2118, Tukey's multiple comparisons test: Static + Sham vs. Static + UL: *p* = 0.0367, Static + UL vs. Run + Sham: *p* = 0.0011, Static + UL vs. Run + UL: *p* = 0.0188 for the percentage of novel arms; two‐way ANOVA: *F*_(1,36)_ = 1.616, *p* = 0.2118, Tukey's multiple comparisons test: Static + UL vs. Run + Sham: *p* = 0.0035, Static + UL vs. Run + UL: *p* = 0.0018 for the total time of the novel arms), Left arm (G) was set as the novel arms (two‐way ANOVA: *F*_(1,36)_ = 0.9306, *p* = 0.3412, Tukey's multiple comparisons test: Static + UL vs. Run + Sham: *p* = 0.0288, Static + UL vs. Run + UL: *p* = 0.0104 for the percentage of novel arms; two‐way ANOVA: *F*_(1,36)_ = 3.727, *p* = 0.0.15, Tukey's multiple comparisons test: Static + Sham vs. Static + UL: *p* = 0.0325, Static + UL vs. Run + Sham: *p* = 0.0002, Static + UL vs. Run + UL: *p* = 0.0003 for the total time of the novel arms, *n* = 10/group in T‐maze). (H) Aerobic exercise significantly improves the number of errors in UL‐induced reference memory. (two‐way ANOVA: *F*_(27,324)_ = 1.766, *p* = 0.0122, Tukey's multiple comparisons test: Sham + Static vs. UL + Static: *p* = 0.0019, UL + Static vs. Sham + Run: *p* = 0.0054, UL + Static vs. UL + Run: *p* < 0.0001). (I) Aerobic exercise significantly reduces the number of errors in working memory induced by UL (two‐way ANOVA: *F*_(27,324)_ = 1.661, *p* = 0.0006, Tukey's multiple comparisons test: Sham + Static vs. UL + Static: *p* = 0.0011, Sham + Static vs. Sham + Run: *p* = 0.0269, UL + Static vs. Sham + Run: *p* < 0.0001, UL + Static vs. UL + Run: *p* < 0.0001). (J) Total time to complete tasks decreased by aerobic exercise. (two‐way ANOVA: *F*_(27,359)_ = 1.012, *p* = 0.4507, Tukey's multiple comparisons test: Sham + Static vs. UL + Static: *p* = 0.0004, UL + Static vs. Sham + Run: *p* < 0.0001, UL + Static vs. UL + Run: *p* < 0.0001, *n* = 10/group). (K) Throughout the training, Sham + Static, Sham + UL, Run + Static, and Run + UL mice froze at a comparable level during the trace interval (two‐way ANOVA, *F*_(3,72)_ = 1.861, *p* = 0.1439). (L, M) Contextual and tone‐cued fear conditioning were improved in running mice (two‐way ANOVA: *F*_(1,36)_ = 3.797, *p* = 0.0592, Tukey's multiple comparisons test: Static + Sham vs. Static + UL: *p* = 0.0018, Static + UL vs. Run + Sham: *p* = 0.0012, Static + UL vs. Run + UL: *p* = 0.0306. two‐way ANOVA: *F*_(1,36)_ = 9.348, *p* = 0.0042, Tukey's multiple comparisons test: Static + Sham vs. Static + UL: *p* = 0.0001, Static + UL vs. Run + Sham: *p* = 0.0045, Static + UL vs. Run + UL: *p* = 0.0285, *n* = 10/group in fear conditioning). *p < 0.05, **p < 0.01, ***p < 0.001, ****p < 0.0001.

Sham and UL mice were randomly divided into running and static groups, respectively. The running groups underwent low‐speed adaptive treadmill training in the first week, followed by high‐speed aerobic exercise for the subsequent 3 weeks, while the static groups were placed on a stationary treadmill without any exercise intervention (Figure 3A). In the open‐field test, there were no significant differences in total distance traveled, average movement speed, stationary time, or time spent in the center among the Sham + Static, UL + Static, Sham + Run, and UL + Run groups (Figure 3B–E). Compared with the Sham + Static group, the UL + Static group exhibited significant cognitive impairments in spatial short‐term memory (T‐maze), working memory and spatial long‐term memory (Radial 8‐Arm Maze), and associative long‐term memory (fear conditioning test; Figure 3F–M). Aerobic exercise did not affect these cognitive functions when comparing Sham + Static with Sham + Run. However, in the T‐maze task, UL + Run mice exhibited significantly more entries and spent more time in the novel arm (Figure 3F,G). In the Radial 8‐Arm Maze task, they also made fewer entry errors (Figure 3H,I) and completed the task in less time than UL + Static mice (Figure 3J). These results indicate that aerobic exercise improved spatial short‐term and working memory impairment induced by UL. For long‐term memory, both contextual and tone‐cued fear conditioning responses were increased in the running group compared to the static group (Figure 3K–M), suggesting that aerobic exercise can improve long‐term memory in UL mice.

### 3.4 Aerobic Exercise Promotes Neurogenesis of DG After UL

**FIGURE 4:**
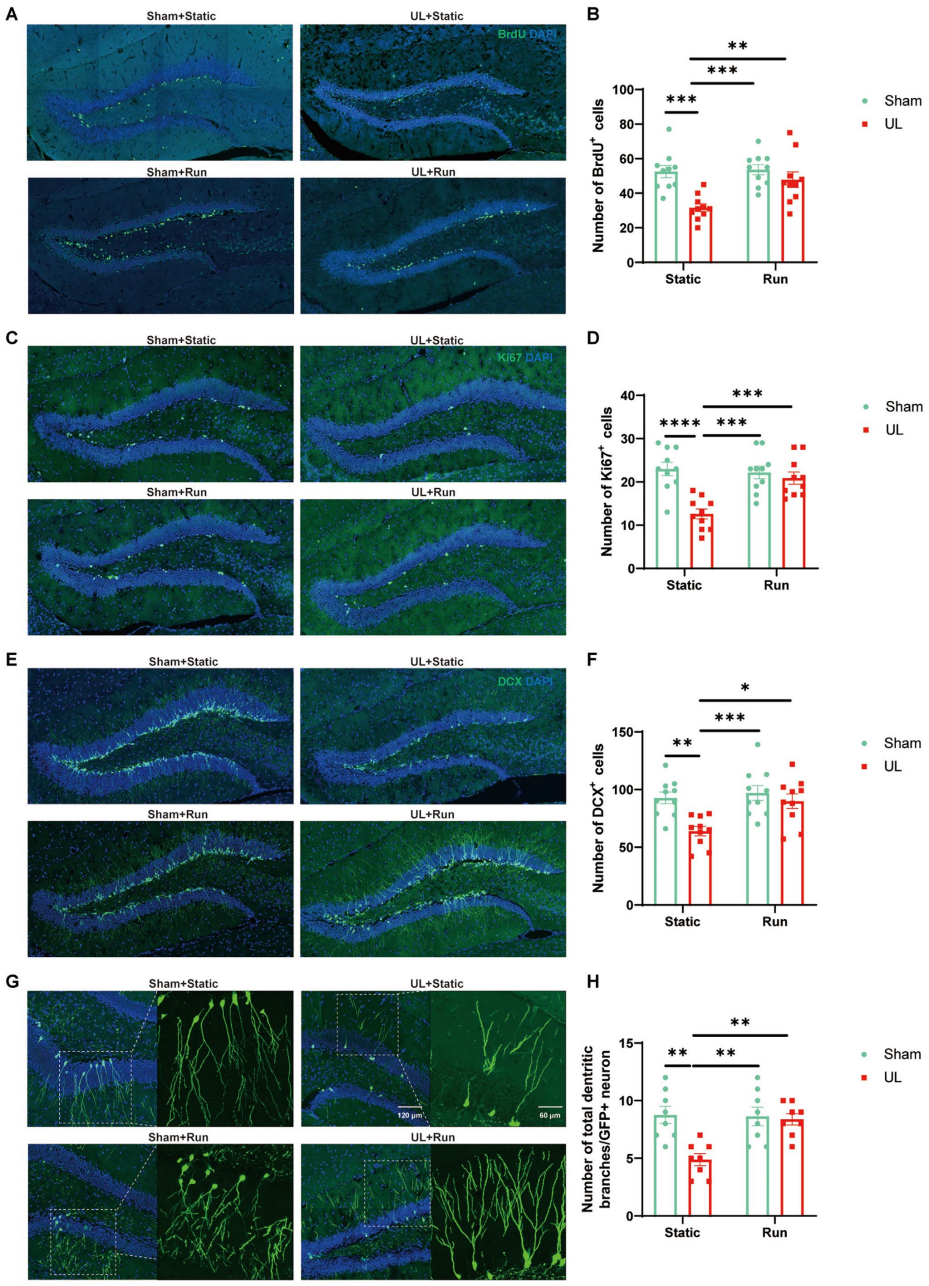
Increased adult neurogenesis after aerobic exercise in the DG. (A) Representative images showing BrdU+ and DAPI+ cells in the DG. (B) The number of BrdU+ cells in the DG increased in running mice. (two‐way ANOVA: *F*_(1,36)_ = 4.897, *p* = 0.0333, Tukey's multiple comparisons test: Static + Sham vs. Static + UL: *p* = 0.0006, Static + UL vs. Run + Sham: *p* = 0.0003, Static + UL vs. Run + UL: *p* = 0.0097, ten mice per group and five sections per mouse). (C) Representative images showing Ki67+ and DAPI+ cells in the DG. (D) The number of Ki67+ cells in the DG increased in running mice. (two‐way ANOVA: *F*_(1,36)_ = 10.57, *p* = 0.0025, Tukey's multiple comparisons test: Static + Sham vs. Static + UL: *p* < 0.0001, Static + UL vs. Run + Sham: *p* = 0.0001, Static + UL vs. Run + UL: *p* = 0.0009, ten mice per group and five sections per mouse). (E) Representative images showing DCX+ and DAPI+ cells in the DG. (F) The number of DCX+ cells in the DG increased in running mice (two‐way ANOVA: *F*_(1,36)_ = 3.821, *p* = 0.0584, Tukey's multiple comparisons test: Static + Sham vs. Static + UL: *p* = 0.0042, Static + UL vs. Run + Sham: *p* = 0.0009, Static + UL vs. Run + UL: *p* = 0.0113, ten mice per group and five sections per mouse). (G) Representative photomicrographs of the dendritic branches of GFP+ cells in the DG. GFP+ cells were labeled with retrovirus pROVEF1a‐EGFP, which was injected in the DG. (H) The total number of dendritic branches of one GFP+ neuron increased in running mice (two‐way ANOVA: *F*_(1,28)_ = 7.655, *p* = 0.0099, Tukey's multiple comparisons test: Static + Sham vs. Static + UL: *p* = 0.0014, Static + UL vs. Run + Sham: *p* = 0.0020, Static + UL vs. Run + UL: *p* = 0.0040, eight mice per group, five sections per mouse, and 8–10 cells per section).

Compared with the Sham + Static group, the UL + Static group exhibited marked impairments in hippocampal neurogenesis, as reflected by significant reductions in the numbers of BrdU‐, Ki67‐, and DCX‐positive cells, as well as a reduction in the total number of dendritic branches in the DG (Figure 4A–H). Following aerobic exercise intervention, these neurogenic deficits were markedly ameliorated in UL + Run mice, with all indicators increasing to levels comparable to those of the Sham groups (Figure 4A–H), suggesting that running effectively reverses UL‐induced neurogenic impairments. Notably, no significant differences in these cellular indices were observed between the Sham + Static and Sham + Run groups (Figure 4A–H). However, when integrated with the behavioral findings, the Sham + Run group displayed significantly enhanced working memory performance relative to the Sham + Static group, further supporting that running exerts a genuine physiological benefit rather than reflecting nonspecific effects such as handling or environmental enrichment.

### 3.5 Differential Gene Expression Analysis of the DG in Sham, UL, and UL + Running Groups

**FIGURE 5:**
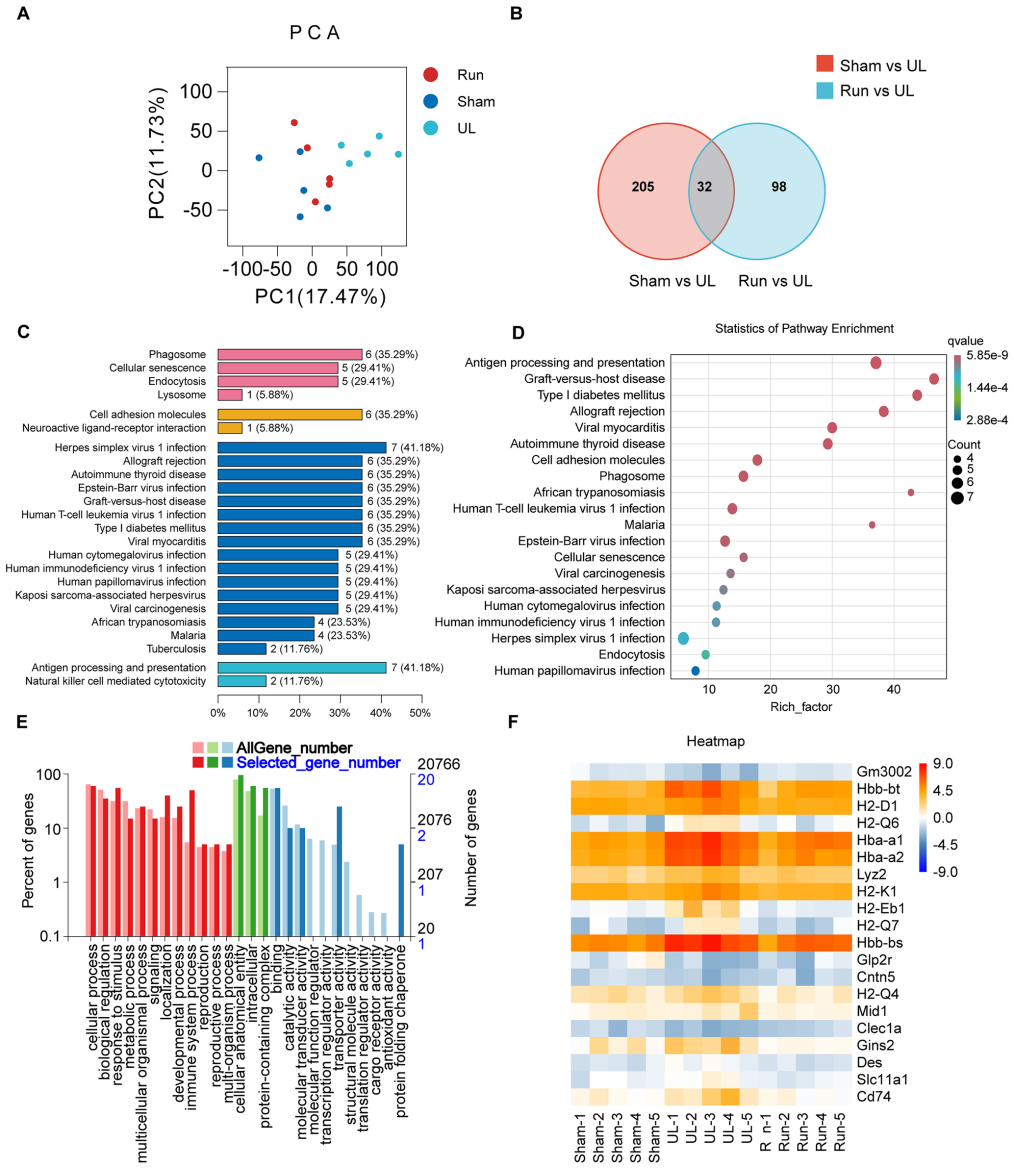
Differential expression gene (DEG) analysis of sham, UL, and running group. (A) Principal component analysis (PCA) of gene expression in Sham, UL and UL + Runing groups. (B) Venn diagram clearly shows the overlapping and unique DEGs between the different DG samples: Sham 28d vs UL 28d, Running 28d vs UL 28d. A total of 32 identical differential genes. (C) KEGG classification was enriched in Cell adhesion molecules, antigen processing and presentation of environmental information processing and autoimmune‐related human diseases. (D) KEGG bubble plot mainly enriched in pathways related to antigen processing and presentation, neuroimmune, and inflammatory related diseases. (E) GO classification was mainly enriched in cellular processes, cellular anatomical entities, and binding. (F) Heatmap of DEGs showed significant changes in neuroimmune and inflammatory related pathways (*n* = 5/group in RNA‐seq).

To elucidate the mechanisms underlying the cognitive improvement in running mice following UL, we performed RNA sequencing (RNA‐seq) analysis on the hippocampal DG. Our results showed that gene expression profiles in the UL group were mainly located at the positive end of PC1 and PC2, while those in the Sham and UL + Run groups were mainly located at the negative end of PC1 and PC2, suggesting a high degree of similarity in gene expression between the Sham and UL + Run groups (Figure 5A). Venn diagrams illustrated the DEGs among the Sham, UL, and UL + Run groups (Figure 5B). A total of 237 genes were found to be significantly differentially expressed between the Sham and UL groups, while 130 genes were differentially expressed between the UL + Run and UL groups. Among these, 32 genes were commonly differentially expressed in both the Sham vs. UL and UL + Run vs. UL comparisons, and expression changes in 23 of these 32 genes observed between the Sham and UL groups were reversed in the UL + Run group. Kyoto Encyclopedia of Genes and Genomes (KEGG) pathway analysis of these 23 genes identified several significantly enriched pathways, including those related to environmental information processing, such as “Cellular senescence”, “Cell adhesion molecules”, and “Neuroactive ligand‐receptor interaction”, as well as pathways related to human diseases and organismal systems, such as “Antigen processing and presentation”, “Graft‐versus‐host disease”, “Allograft rejection”, and “Phagosome” (Figure 5C,D). Gene Ontology (GO) analysis also suggested that the DEGs following UL and UL + Run interventions were predominantly enriched in pathways associated with immune and neuroinflammation functions (Figure 5E). Among the 23 DEGs, 17 exhibited increased expression following UL and decreased expression following aerobic exercise, while 6 showed decreased expression following UL and increased expression following aerobic exercise, as illustrated by the clustered heatmap (Figure 5F). Given the suppression of neurogenesis and cognitive dysfunction observed in UL mice, and the promotion of neurogenesis and cognitive improvement by aerobic exercise, we speculate that alterations in neuroinflammation and the immune microenvironment may underlie the changes in neurogenesis and subsequent cognitive function.

### 3.6 qRT‐PCR Validation and Hypothesis

**FIGURE 6:**
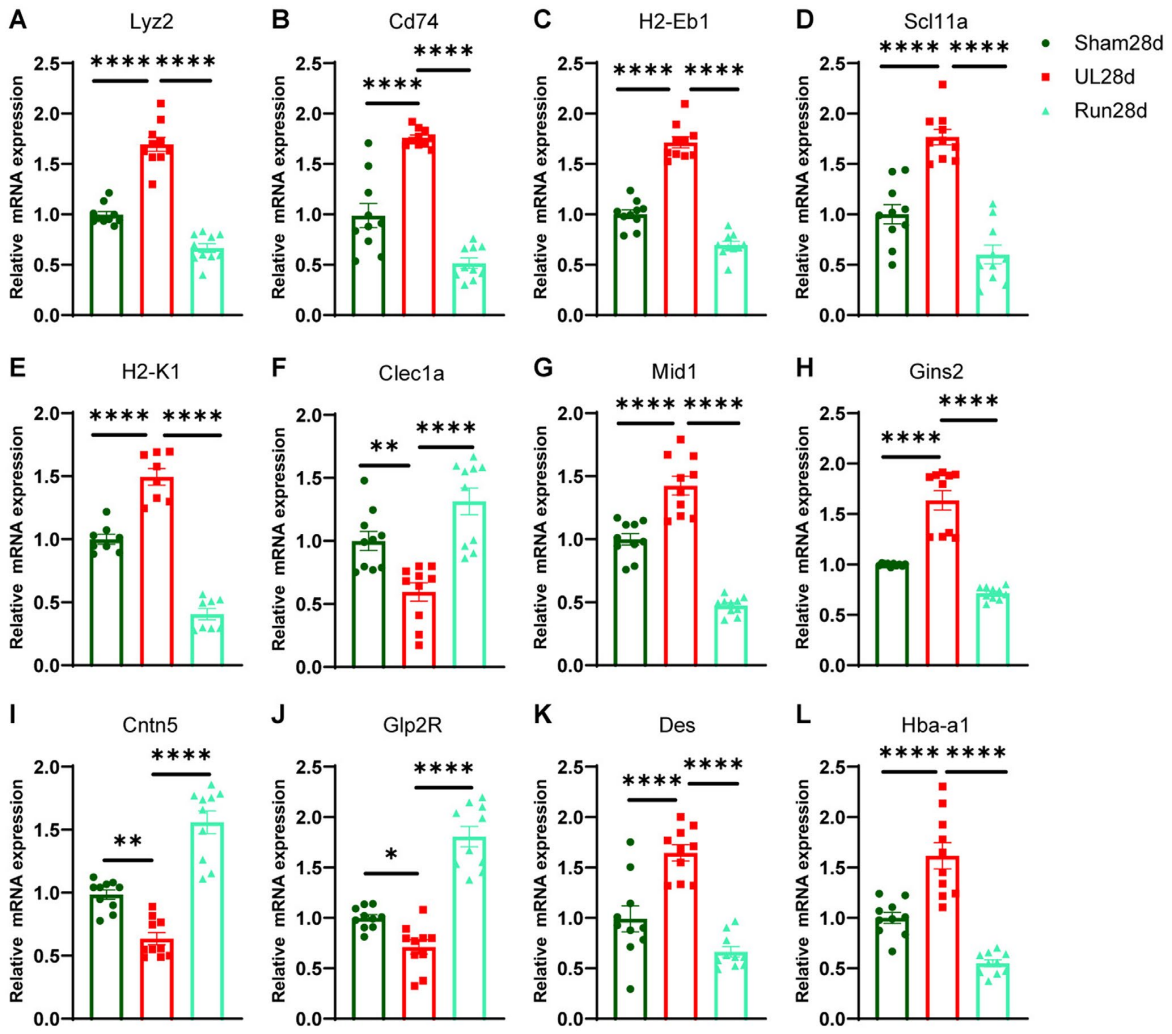
qRT‐PCR validation of gene expression changes related to cognitive function. (A–E, G, H, K, L) The *Lyz2* (Ordinary one‐way ANOVA, *F*_(2,27)_ = 102.0, *p* < 0.0001), *Cd74* (Ordinary one‐way ANOVA, *F*_(2,27)_ = 60.91, *p* < 0.0001), *H2‐Eb1* (Ordinary one‐way ANOVA, *F*_(2,27)_ = 136.9, *p* < 0.0001), *Slc11a1* (Ordinary one‐way ANOVA, *F*_(2,27)_ = 40.07, *p* < 0.0001), *H2‐K1* (Ordinary one‐way ANOVA, *F*_(2,27)_ = 90.56, *p* < 0.0001), *Mid1* (Ordinary one‐way ANOVA, *F*_(2,27)_ = 61.00, *p* < 0.0001), *Gins2* (Ordinary one‐way ANOVA, *F*_(2,27)_ = 65.38, *p* < 0.0001), *Des* (Ordinary one‐way ANOVA, *F*_(2,27)_ = 26.82, *p* < 0.0001), *Hba‐a1* (Ordinary one‐way ANOVA, *F*_(2,27)_ = 35.63, *p* < 0.0001) genes were up‐regulated at 28d after UL and down‐regulated by aerobic exercise. (F, I, J) The *Clec1a* (Ordinary one‐way ANOVA, *F*_(2,27)_ = 17.63, *p* = 0.0002), *Cntn5* (Ordinary one‐way ANOVA, *F*_(2,27)_ = 50.28, *p* < 0.0001), *Glp2R* (Ordinary one‐way ANOVA, *F*_(2,27)_ = 58.78, *p* < 0.0001) genes were down‐regulated at 28d after UL and up‐regulated by aerobic exercise (*n* = 10/group in qRT‐PCR).

To further validate the findings from the clustering heatmap analysis, we extracted mRNA from the hippocampal DG and conducted qRT‐PCR to analyze gene expression levels. The results demonstrated that the expression of the Lyz2, CD74, Slc11a1, H2‐Eb1, and H2‐K1 genes, which play an important role in antigen presentation and immune regulation, increased after UL and decreased after aerobic exercise, while the expression of the Clec1a gene decreased after UL and increased after aerobic exercise (Figure 6A–F). Importantly, the expression changes of four cell proliferation and neurodevelopment‐related genes in the UL group, Gins2, Mid1, Cntn5, and Glp2r, were reversed by aerobic exercise (Figure 6G–J). Interestingly, the expression of the intermediate fibrin gene Des and the hemoglobin subunit alpha 1 (Hba‐a1) gene was increased after UL and decreased after aerobic exercise, suggesting the involvement of the cellular matrix and the redox environment (Figure 6K,L).

### 3.7 AZD1480 Ameliorates UL‐Induced Cognitive Dysfunction, Whereas LPS Significantly Counteracts the Cognitive Improvements Conferred by Running

**FIGURE 7:**
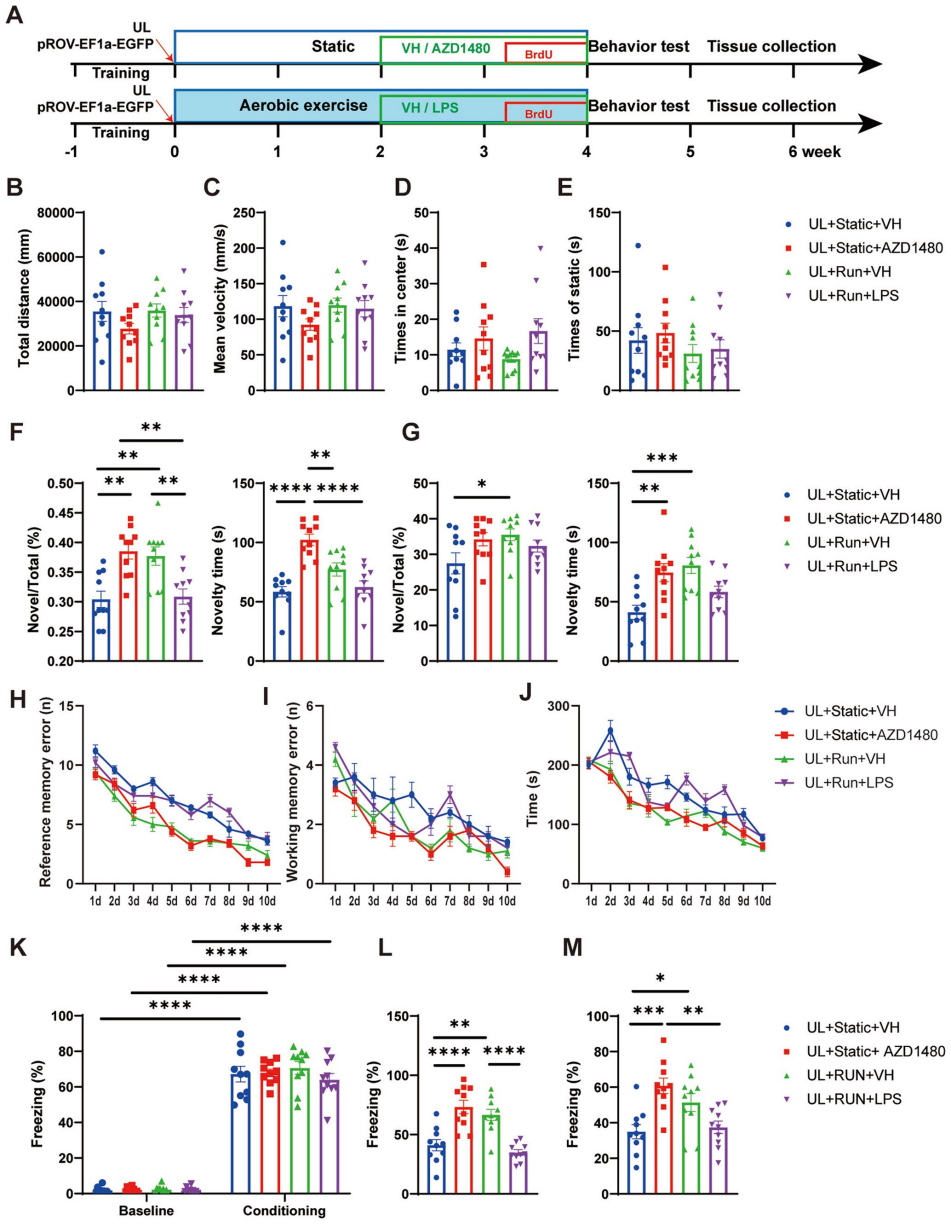
AZD1480 ameliorates UL‐induced cognitive dysfunction, whereas LPS significantly counteracts the cognitive improvements conferred by running. (A) Schematic of the experimental design showing AZD1480 and LPS interventions in UL mice. (B, C) In the open field test, there were no significant differences in total movement distance and average speed among the four groups of mice: UL + Static + VH, UL + Static + AZD1480, UL + Run + VH, UL + Run + LPS (One‐way ANOVA: *F*_(3,36)_ = 1.236, *p* = 0.3110, and *F*_(3,36)_ = 1.257, *p* = 0.3037). (D, E) There were no significant intergroup differences in terms of total immobility time and the duration spent in the central zone of the open field for the UL + Static + VH, UL + Static + AZD1480, UL + Run + VH, UL + Run + LPS groups (One‐way ANOVA: *F*_(3,36)_ = 1.780, *p* = 0.1684 and *F*_(3,36)_ = 0.7831, *p* = 0.5112, *n* = 10/group in OFT). (F, G) AZD1480 ameliorated the UL‐induced working memory impairment, whereas LPS counteracts the cognitive improvements conferred by running, manifested by the total entry to and time spent in the novel arm. Right arm (F) was set as the novel arm (One‐way ANOVA: *F*_(3,36)_ = 9.760, *p* < 0.0001), Tukey's multiple comparisons test: UL + Static + VH vs. UL + Static + AZD1480: *p* = 0.0011, UL + Static + VH vs. UL + Run + VH: *p* = 0.0036, UL + AZD1480 vs. UL + Run + LPS: *p* = 0.0022, UL + Run + VH vs. UL + Run + LPS: *p* = 0.0068 for the percentage of novel arms; One‐way ANOVA: *F*_(3,36)_ = 15.93, *p* < 0.0001, Tukey's multiple comparisons test: UL + Static + VH vs. UL + Static + AZD1480: *p* < 0.0001, UL + AZD1480 vs. UL + Run + VH: *p* = 0.0056, UL + Static + AZD1480 vs. UL + Run + LPS: *p* < 0.0001 for the total time of the novel arms, Left arm (G) was set as the novel arms (One‐way ANOVA: *F*_(3,36)_ = 2.814, *p* = 0.0529, Tukey's multiple comparisons test: UL + Static + VH vs. UL + Run + VH *p* = 0.0488, for the percentage of novel arms; One‐way ANOVA: *F*_(3,36)_ = 7.771, *p* = 0.0004, Tukey's multiple comparisons test: UL + Static + VH vs. UL + Static + AZD1480: *p* = 0.0036, UL + Static + VH vs. UL + Run+ VH: *p* = 0.0005 for the total time of the novel arms, *n* = 10/group in T maze). (H) AZD1480 significantly improves the number of errors in UL‐induced reference memory, whereas LPS counteracts the cognitive improvements conferred by running (two‐way ANOVA: *F*_(27,324)_ = 2.115, *p* = 0.0013, Tukey's multiple comparisons test: UL + Static + VH vs. UL + Static + AZD1480: *p* < 0.0001, UL + Static + VH vs. UL + Run + VH: *p* < 0.0001, UL + Static + AZD1480 vs. UL + Run + LPS: *p* < 0.0001, UL + Run + VH vs. UL + Run + LPS: *p* < 0.0001). (I) AZD1480 significantly reduces the number of errors in working memory induced by UL (two‐way ANOVA: *F*_(27,324)_ = 1.623, *p* = 0.0283, Tukey's multiple comparisons test: UL + Static + VH vs. UL + Static + AZD1480: *p* < 0.0001, UL + Static + VH vs. UL + Run + VH: *p* = 0.0273, UL + Static + AZD1480 vs. UL + Run + LPS: *p* = 0.0006). (J) Total time to complete tasks decreased by aerobic exercise (two‐way ANOVA: *F*_(27,324)_ = 3.394, *p* < 0.0001), Tukey's multiple comparisons test: UL + Static + VH vs. UL + Static + AZD1480: *p* = 0.0003, UL + Static + VH vs. UL + Run + VH: *p* = 0.0003, UL + Static + AZD1480 vs. UL + Run + LPS: *p* = 0.0001, UL + Run + VH vs. UL + Run + LPS: *p* = 0.0001, *n* = 10/group, data are presented as mean ± standard error of the mean (SEM). (K) Throughout the training, UL + Static + VH, UL + Static + AZD1480, UL + Run + VH and UL + Run + LPS mice froze at comparable level during the trace interval (two‐way ANOVA, *F*_(3,72)_ = 0.5261, *p* = 0.6658). (L, M) AZD1480 and running resulted in improved contextual and tone‐cued fear conditioning, but LPS prevented the running‐induced improvement (One‐way ANOVA: *F*_(3,36)_ = 17.34, *p* < 0.0001, Tukey's multiple comparisons test: UL + Static + VH vs. UL + Static + AZD1480: *p* < 0.0001, UL + Static + VH vs. UL + Run + VH: *p* = 0.0017, UL + Static + AZD1480 vs. UL + Run + LPS: *p* < 0.0001, UL + Run + VH vs. UL + Run + LPS: *p* < 0.0001. One‐way ANOVA: *F*_(3,36)_ = 8.139, *p* = 0.0003, Tukey's multiple comparisons test: UL + Static + VH vs. UL + Static + AZD1480: *p* = 0.0007, UL + Static + VH vs. UL + Run + VH: *p* = 0.0462, UL + Static + AZD1480 vs. UL + Run + LPS: *p* = 0.0023, *n* = 10/group in fear conditioning). *p < 0.05, **p < 0.01, ***p < 0.001, ****p < 0.0001.

UL mice were randomly assigned to four groups: UL + Static + VH, UL + Static + AZD1480, UL + Run + VH, and UL + Run + LPS. Mice in the static groups were placed on a stationary treadmill; after 2 weeks, one group received AZD1480 while the other received the DMSO vehicle, with no exercise intervention. Mice in the running group underwent low‐speed adaptive treadmill training in the first week, followed by 3 weeks of high‐speed aerobic exercise. During the final 2 weeks, one subgroup was injected with LPS while the other received saline control (Figure 7A). The open field test revealed no significant differences in total distance traveled, average speed, immobility time, or time spent in the center among the four UL groups (Figure 7B–E). In the T‐maze test, the UL + Static + AZD1480 and UL + Run + VH groups entered the novel arm significantly more frequently and spent more time there, whereas LPS injection abolished the exercise‐induced improvement (Figure 7F,G). In the radial arm maze test, the UL + Static + AZD1480 and UL + Run + VH groups made fewer errors (Figure 7H,I) and required less time to complete the task (Figure 7J) compared to the UL + Static + VH group. The UL + Run + LPS group showed no significant difference in errors or time compared to the UL + Static + VH group. In long‐term memory tests, both the UL + Static + AZD1480 and UL + Run + VH groups exhibited enhanced contextual and cued fear conditioning responses compared to the UL + Static + VH and UL + Run + LPS groups (Figure 7K–M). The JAK1/JAK2 inhibitor AZD1480 reduced the expression of neuroinflammation‐related genes, whereas LPS promoted their expression (Figure S1). AZD1480 inhibits the JAK1/JAK2 pathway, leading to suppressed expression of MHC class II‐related inflammatory factors (CD74, H2‐Eb1) and reduced release of inflammatory cytokines [35]; this is consistent with our observations. Aerobic exercise ameliorates UL‐induced impairments in short‐term spatial, working, and long‐term memory. Similarly, AZD1480 exerts an equivalent protective effect by suppressing neuroinflammatory responses. Conversely, administration of LPS in exercised mice abolished the running‐mediated protective effects.

### 3.8 AZD1480 Rescued the UL‐Induced Deficit in Neurogenesis, Whereas LPS Prevented the Running‐Induced Amelioration

**FIGURE 8:**
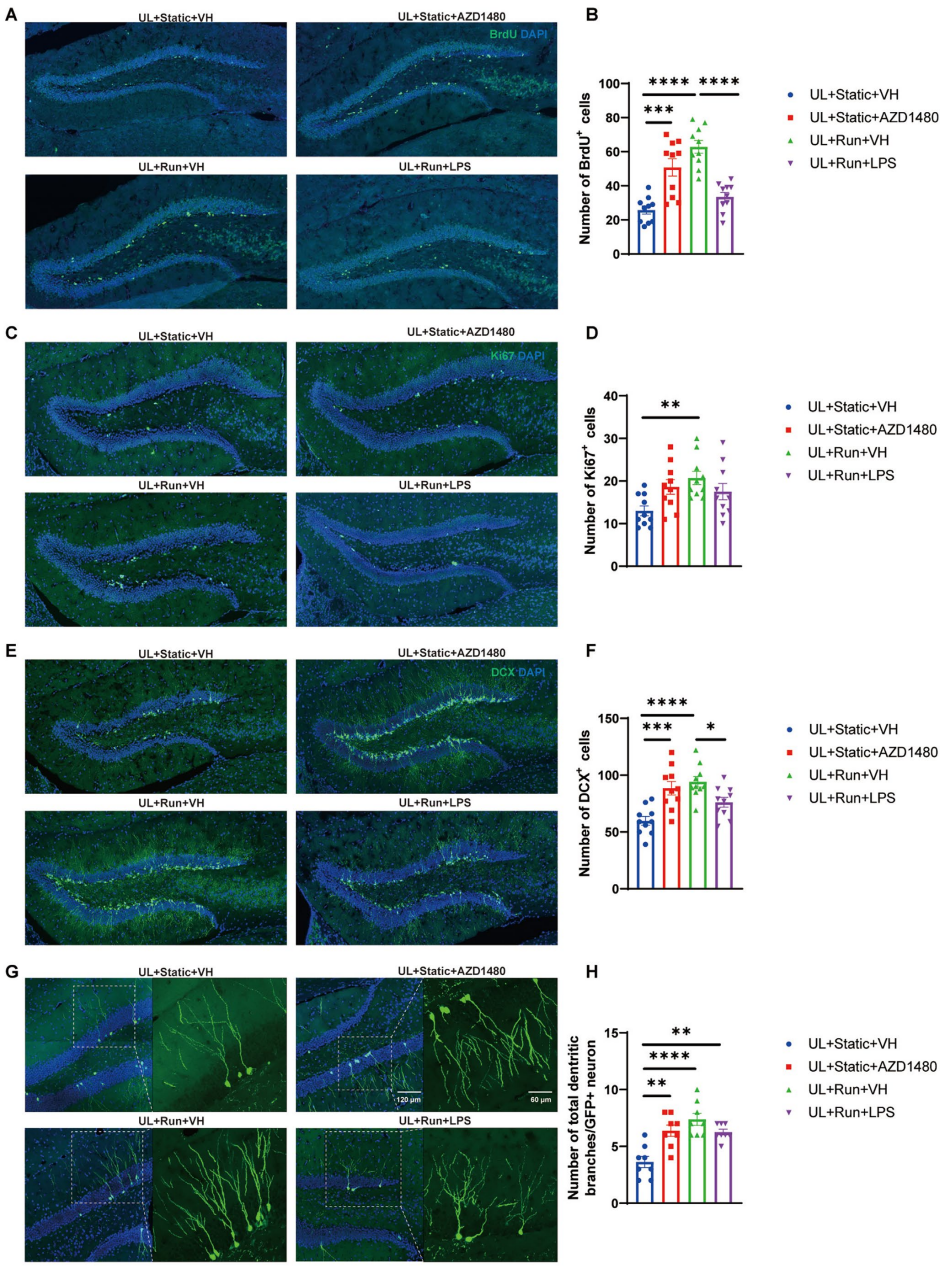
AZD1480 rescues the UL‐mediated suppression of hippocampal neurogenesis, while LPS markedly attenuates the pro‐proliferative effects of running. (A) Representative images showing BrdU+ and DAPI+ cells in the DG. (B) AZD1480 increases the number of BrdU+ cells induced by UL, while LPS counteracts the neuroproliferative effects induced by running. (One‐way ANOVA: *F*_(3,36)_ = 21.29, *p* < 0.0001, Tukey's multiple comparisons test: UL + Static + VH vs. UL + Static + AZD1480: *p* = 0.0001, UL + Static + VH vs. UL + Run + VH: *p* < 0.0001, UL + Static + AZD1480 vs. UL + Run + LPS: *p* = 0.0094, UL + Run + VH vs. UL + Run + LPS: *p* < 0.0001). (C) Representative images showing Ki67+ and DAPI+ cells in the DG. (D) Running increases the number of UL‐induced Ki67+ cells, while AZD1480 and LPS have no significant effect on Ki67+ cell counts. (One‐way ANOVA: *F*_(3,36)_ = 4.058, *p* = 0.0139, Tukey's multiple comparisons test: UL + Static + VH vs. UL + Run + VH: *p* = 0.0092). (E) Representative images showing DCX+ and DAPI+ cells in the DG. (F) AZD1480 increases the number of DCX+ cells induced by UL, while LPS counteracts the neuroproliferative effects induced by running. (One‐way ANOVA: *F*_(3,36)_ = 10.35, *p* < 0.0001, Tukey's multiple comparisons test: UL + Static + VH vs. UL + Static + AZD1480: *p* = 0.0007, UL + Static + VH vs. UL + Run + VH: *p* < 0.0001, UL + Run + VH vs. UL + Run + LPS: *p* = 0.0490). (G) Representative photomicrographs of the dendritic branches of GFP+ cells in the DG. GFP+ cells were labeled with retrovirus pROVEF1a‐EGFP, which was injected in the DG. (H) AZD1480 significantly increases the total number of dendritic branches in individual GFP^+^ newborn neurons induced by UL. (One‐way ANOVA: *F*_(3,28)_ = 12.20, *p* < 0.0001, Tukey's multiple comparisons test: UL + Static + VH vs. UL + Static + AZD1480: *p* = 0.0012, UL + Static + VH vs. UL + Run + VH < 0.0001, UL + Static + VH vs. UL + RUN + LPS: *p* = 0.0020).

Compared to the UL + Static + VH group, the running group and the AZD1480‐treated group showed a significantly higher number of BrdU‐, Ki67‐, and DCX‐positive cells in the hippocampal dentate gyrus (Figure 8A–F). LPS administration abolished the running‐induced increase in BrdU‐positive and DCX‐positive cells, but had no significant effect on Ki67‐positive cells. Furthermore, the total dendritic branch points were significantly increased in the UL + Static + AZD1480, UL + Run + VH, and UL + Run + LPS groups compared to UL + Static + VH (Figure 8G,H). These results indicate that aerobic exercise promotes neurogenesis in UL mice. By inhibiting the JAK1/JAK2 pathway and modulating related gene expression (Figure S1), AZD1480 reduces the release of inflammatory factors, thereby mimicking the effects of aerobic exercise and improving neuronal proliferation and differentiation in mice. LPS‐induced neuroinflammation suppresses neuronal proliferation but does not significantly affect neuronal differentiation.

## 4 Discussion

Our study shows for the first time that aerobic exercise reverses UL‐induced cognitive dysfunction and hippocampal neurogenesis impairment. The behavioral results indicated that spatial memory deficits at different time scales in UL model mice, as well as tone‐cued long‐term memory impairments, were effectively rescued by aerobic exercise. Further histological analysis showed that proliferation and differentiation deficits of DG neural stem cells were also reversed by aerobic exercise in UL model mice. Further studies revealed that the altered immune microenvironment in the hippocampus may be a key factor mediating the rehabilitation effects of aerobic exercise.

Our previous study showed that cognitive deficits remained at 28 days after UL, despite vestibular malfunction‐related phenotypes being significantly recovered after 7 days [38]. Additionally, clinical studies reported the association between vestibular loss and dementia and even AD, suggesting the importance of immediate intervention against cognitive dysfunction. As one of the most common physical therapies, aerobic exercise improves cognition via mechanisms such as metabolic reprogramming, adult neurogenesis, and neural inflammation inhibition. Previously, we also observed the hyperactivation of neurons in the granular layer of DG within 7 days, suggesting the involvement of DG in UL‐induced cognitive impairments. DG is the primary brain region that maintains neural stem cells in adult animals, and damage to neurogenesis in the DG impairs spatial cognition and reduces hippocampal volume [39, 40, 41], as observed in vestibular disorder patients.

To prove the hypothesis that the impairment of adult neurogenesis mediates UL‐induced cognitive dysfunction, we evaluate the protective effect of aerobic exercise on DG neurogenesis and spatial memory. OFT results revealed no significant changes in center zone duration either after UL induction or following aerobic exercise, suggesting that the observed behavioral alterations are unlikely to be associated with anxiety/depression‐like states. However, aerobic exercise improved all short and long‐term cognitive‐related functions and neurogenesis in DG 28 days after UL. During stem cells integration into the DG neural network, neural stem cells may facilitate spatial cognition through pattern separation, potentially contributing to the recovery of long‐term spatial memory following UL [42, 43, 44]. Although promoting neurogenesis may enhance short‐term spatial memory through mechanisms such as neural circuit oscillations, it cannot be ruled out that aerobic exercise may also influence short‐term spatial memory through other pathways, including metabolic effects [33, 45, 46].

To clarify the underlying mechanisms, we employed RNA‐seq to screen the pathways related to aerobic exercise protected effects against UL‐induced cognitive dysfunction. Further qRT‐PCR validation indicated that 3 pathways may be specifically affected: (1) MHC class II–related molecules (H2‐Eb1 and CD74), which mediate antigen presentation and microglial activation [47], were markedly upregulated after UL but reduced by aerobic exercise, suggesting that exercise may attenuate excessive MHC II–driven neuroinflammation and thereby supports a pro‐neurogenic hippocampal milieu; (2) MHC class I molecules (H2‐K1 and H2‐Q family members), known to regulate endogenous antigen presentation and modulate neuronal synaptic remodeling and plasticity [48, 49] were similarly elevated by UL and normalized by exercise, indicating that aerobic training may restore adaptive MHC I–dependent synaptic dynamics to facilitate newborn neuron integration; (3) Neurodevelopmental and structural genes (Cntn5 and Mid1) govern cell–cell interactions, axon outgrowth, and cytoskeletal organization required for circuit formation and neuronal maturation [50, 51, 52]. They were suppressed after UL but rescued by exercise, consistent with enhanced structural remodeling that supports the maturation and functional incorporation of adult‐born neurons.

To demonstrate the necessity and sufficiency of key candidate genes in mediating the exercise‐induced effects, we performed bidirectional pharmacological modulation using LPS and AZD1480, followed by assessments of neurogenesis and cognitive outcomes. On the one hand, LPS‐induced changes in the expression of genes related to the M1/M2 phenotypes (including H2‐Eb1 and CD74) have been widely used as a model of neuroinflammation [53, 54, 55]. On the other hand, previous literature reports that AZD1480 treatment can reduce cell numbers and cluster‐specific expression of the antigen‐presentation genes H2‐Eb1 and CD74 and suppress the activation and infiltration of innate and adaptive cells, thereby reducing neuroinflammation [35]. Our results demonstrated that AZD treatment significantly improved behavioral performance and neurogenesis in UL mice, confirming that inhibiting neuroinflammatory signaling can mimic key features of exercise‐induced effects. However, the UL + Run + LPS group showed attenuated cognitive and neurogenic improvements compared with the UL + Run + VH group, indicating that LPS attenuates the beneficial effects of aerobic exercise. These findings provide evidence that neuroinflammation‐related genes identified in our RNA‐seq analysis are mechanistically involved in mediating exercise‐induced recovery.

This study also has certain limitations that warrant consideration. The absence of protein‐level validation restricts the extent to which the transcriptomic alterations identified here can be linked to specific cell populations or functional pathways. Moreover, the lack of quantitative running‐performance metrics precludes examination of potential dose–response relationships between exercise intensity and behavioral or neurogenic outcomes. Future work integrating molecular, cellular, and performance‐level assessments will be necessary to comprehensively delineate the mechanistic basis of exercise‐mediated benefits following UL.

## 5 Conclusion

In summary, we established a UL model to study cognitive impairment caused by vestibular dysfunction. Using behavioral tests, we confirmed that mice exhibited significant cognitive impairment 28 days after UL, accompanied by suppressed hippocampal neurogenesis, which may be a key factor contributing to cognitive dysfunction. Based on these findings, we designed aerobic exercise as a rehabilitation intervention and found that it effectively promoted neurogenesis in UL mice and significantly improved their cognitive dysfunction. In addition, RNA‐seq analysis identified key molecular processes associated with neurogenesis, providing clues to further elucidate the underlying mechanisms. Future research should delve deeper into these identified pathways to comprehensively elucidate the upstream and downstream cascades of DEGs. These findings not only deepen our understanding of the neurobiological mechanisms underlying cognitive impairment after UL, but also highlight the urgent need for early cognitive screening and diagnosis in clinical patients with vestibular dysfunction. Implementation of early cognitive screening in these patients may significantly improve the accuracy of disease severity assessment, optimize diagnostic precision and guide personalized treatment strategies. Ultimately, more effective management of vestibular dysfunction and associated cognitive impairment, combined with therapeutic interventions such as vestibular rehabilitation, has the potential to significantly improve patients' quality of life.

## Author Contributions

S.Z., Y.L., J.W. conceived the study. S.Z., Z.Z., X.Y., and Y.L. designed the experiments. Z.Z., X.Y., E.T., and Y.Z. performed the experiments and analyzed the data. Z.Z. prepared the initial draft of the manuscript. Z.G., J.C., J.G., S.S., W.X., N.Z., and C.Q. reviewed and edited the manuscript. All authors approved the final manuscript.

## Funding

This work was supported by grants from the National Key Research and Development Program of China (2023YFC2508002 and 2023YFC2508403), the National Natural Science Foundation of China (82371168), the Young Scientists Fund of the National Natural Science Foundation of China (82501420), and Jiangxi Provincial Natural Science Foundation Youth Project (20252BAC200112).

## Ethics Statement

The animal study protocol was approved by the Animal Care and Use Committee of Huazhong University of Science and Technology (approval code: 3971).

## Conflicts of Interest

The authors declare no conflicts of interest.

## Supporting information


**Figure S1:** Effect of AZD1480 and LPS on gene expression by q‐PCR.

## Data Availability

The datasets used and/or analyzed during the current study are available from the corresponding author on reasonable request.
