# Supplementary material for: Aerobic Exercise Promotes Hippocampal Neurogenesis and Ameliorates Cognitive Dysfunction Induced by Unilateral Labyrinthectomy
Source: CNS Neurosci Ther. 2026 Feb 3;32(2):e70773. doi: 10.1002/cns.70773 (PMC12865327; doi:10.1002/cns.70773)
Supplement: Supplementary file 1 — Figure S1: Effect of AZD1480 and LPS on gene expression by q‐PCR. [file CNS-32-e70773-s001.docx]

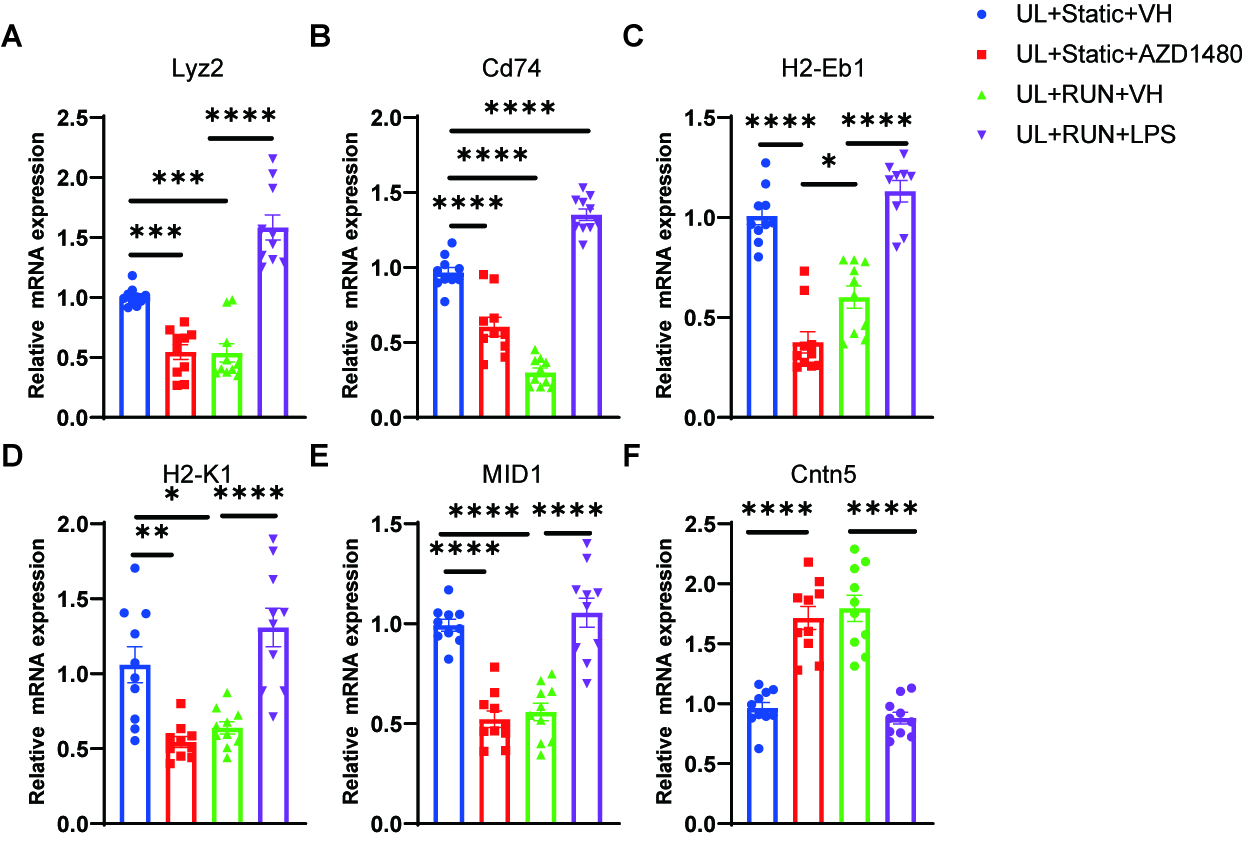


Supporting information

Figure S1. Effect of AZD1480 and LPS on gene expression by q-PCR.

(A-E) AZD1480 administration resulted in the upregulation of genes in UL-induced mice, whereas LPS caused a reversal of the running-induced upregulation of related genes.

*Lyz2* (Ordinary one-way ANOVA, *F* _(3, 36)_ = 46.71, *p* < 0.0001),

*Cd74* (Ordinary one-way ANOVA, *F* _(3, 36)_ = 110.5, *p* < 0.0001),

*H2-Eb1* (Ordinary one-way ANOVA, *F* _(3, 36)_ = 45.56, *p* < 0.0001),

*H2-K1* (Ordinary one-way ANOVA, *F* _(3, 36)_ = 15.31, *p* < 0.0001),

*Mid1* (Ordinary one-way ANOVA, *F* _(3, 36)_ = 32.23, *p* < 0.0001)

(F) AZD1480 administration resulted in the downregulation of genes in UL-induced mice, whereas LPS caused a reversal of the running-induced downregulation of related genes. *Cntn5* (Ordinary one-way ANOVA, *F* _(3, 36)_ = 36.40, *p* < 0.0001).
